# Colorectal Cancer Screening Pilot Project in Tehran-Iran, a Feasibility Study

**DOI:** 10.34172/aim.2023.22

**Published:** 2023-03-01

**Authors:** Hamideh Salimzadeh, Catherine Sauvaget, Alireza Delavari, Anahita Sadeghi, Mohammad Amani, Sepideh Salimzadeh, Azita Karimi, Ali Ghanbari Motlagh, Eric Lucas, Partha Basu, Reza Malekzadeh

**Affiliations:** ^1^Digestive Oncology Research Centre, Digestive Disease Research Institute, Tehran University of Medical Sciences, Tehran, Iran; ^2^Department of Surgery, Institute of Clinical Sciences, Sahlgrenska Academy, University of Gothenburg, Sahlgrenska University Hospital, Östra, 416 85, Gothenburg, Sweden; ^3^Early Detection, Prevention & Infections Branch, International Agency for Research on Cancer, 150 Cours Albert Thomas, 69372 Lyon CEDEX 08, France; ^4^Deputy of Health, Tehran University of Medical Sciences, Tehran, Iran; ^5^Cancer Office, Deputy of Health, Ministry of Health, Tehran, Iran

**Keywords:** Colorectal cancer, Feasibility studies, Screening

## Abstract

**Background:**

Colorectal cancer (CRC) is the third most common cancer in Iran, where there is no organised CRC-screening programme. This study aimed to evaluate feasibility of CRC screening using a qualitative fecal immunochemical test (FIT) among Iranian average-risk adults.

**Methods:**

In this feasibility study, 7039 individuals aged 50–75 years were invited by community health workers (CHWs) in southern Tehran and its suburban districts between April 2018 and November 2019. The CHWs performed a qualitative FIT with cut-off level 50 ng Hb/mL buffer and referred those with positive-FIT for colonoscopy to the endoscopy center of Shariati hospital in Tehran. Outcomes included acceptance rate, FIT positivity rate, colonoscopy compliance, detection rates and positive predictive values (PPVs) with 95% confidence interval for CRC and advanced adenomas (AAs).

**Results:**

Acceptance rate at initial invitation was 71.7%. From 4974 average-risk adults (1600 males and 3374 females) who were offered FIT, 96.8% (n=4813) provided valid samples, of whom 471 (9.8%) tested positive. Among FIT-positive participants, 150 (31.8%) underwent colonoscopy; CRC was detected in 2.0% (n=3) and adenomas in 27.3% (n=41). Detection rate of CRC and AAs per 1000-FIT-screened participants was 0.6 (0.1–1.8) [males: 0.7 (0.01–3.6), females: 0.6 (0.07–2.0)] and 4.2 (2.5–6.4) [males: 5.9 (2.6–11.0), females: 3.4 (1.7–6.0)], respectively. PPVs were 2.0% (0.4–5.7) for CRC and 13.3% (8.3–19.8) for AAs. There was no association between gender and the studied outcomes.

**Conclusion:**

Our results partially support the feasibility of scaling up organized CRC-screening through the existing healthcare system in Iran; it remains to be discussed carefully to ensure the capacity of healthcare system for adequate colonoscopy services.

## Introduction

 Colorectal cancer (CRC) is the third leading cause of cancer deaths with over 1.9 million incident cases annually worldwide.^1^ While the CRC incidence has stabilized over the past few decades among the older population in selected high-income countries thanks to screening,^2,3^ it has been increasing in low- and middle-income countries.^4,5^

 In Iran, CRC with an age-standardized incidence rate of 15.1 per 100 000 person-years is the third most common cancer^6^ and an opportunistic screening-based study showed that the prevalence of colonic adenomas in average-risk Iranians was comparable to those in populations that are considered to have a high incidence of CRCs.^7^ This calls for further investigations for an effective screening strategy and in this regard, stool-base tests such as fecal immunochemical test (FIT) have an acceptable diagnostic yield for CRC in screened populations.^8,9^ Therefore, we assessed the feasibility of FIT-based screening in average-risk Iranians aged 50–75 years with respect to the existing healthcare structure and challenges regarding implementation of CRC screening within the Iranian healthcare system.

## Materials and Methods

###  Study Setting

 This was a feasibility study of the early detection of CRC based on a qualitative FIT. The screening protocol was developed by a joint expert group from the Digestive Diseases Research Institute (DDRI) at Tehran University of Medical Sciences and the International Agency for Research on Cancer (IARC/WHO) in France, considering CRC rates and life expectancy,^10^ efficacy,^11-13^ and cost–effectiveness^14^ of screening in the Iranian context.

 A total of 33 primary healthcare centers (PHCs) located in southern Tehran, Shahr-e-Rey, and Eslamshahr were enlisted in the study, involving their associated community health workers (CHWs). We considered approximately 5000 persons in total (~150 per center) to be screened, assuming 70% uptake rate for FIT^11^ as the main outcome of the study.

 The CHWs, who are often from the village or city they serve and entrusted to provide primary healthcare across the country, were in charge of participant recruitment, conducting interviews, risk-assessment, offering and performing FIT, sending reminder calls, arranging referrals, and administering pre-colonoscopy consultation. The CHWs were paid extra for these tasks as they added to their routine workload. Participants aged 50–75 years were invited either in-person from those who visited the PHC for any other reasons or through telephone to those registered in the Iranian health integrated system (*Samane Iekparche Behdasht*, SIB) held by the Ministry of Health and Medical Education. A written informed consent was obtained from participants after explaining the study objectives and procedures. Average-risk individuals aged 50–75 years who had no documented CRC screening in the last two years were enrolled for FIT screening. We excluded individuals with any of the following criteria from the FIT-screening: inflammatory bowel diseases; a personal history of CRC; family history of CRC in the first-degree relatives; rectal bleeding.^8,9^ Identification of this subgroup referred to as high-risk individuals in this study was done by the CHW using a risk assessment tool and they were referred directly to the endoscopy center at Shariati Hospital in Tehran.

###  Screening Procedure

 Participants were offered a one-step qualitative FIT (*itest, PadyabTeb*, Eshtehard Industrial Estate, Iran) with a cut-off value of 50 ng/mL for detection of hemoglobin in stool which required no dietary restrictions.^15,16^ The CHWs conducted a 25-minute face-to-face interview with each participant to complete the study questionnaire and explain how to obtain stool specimens and return them to the health centers within a maximum of three days after sampling, providing them with an educational pamphlet about stool collection at the end of the interview. One reminder call was sent after one week if the FIT sample was not returned.

 In each PHC, FIT testing was performed by the CHWs on the same day of receiving the samples, following the manufacturer’s instructions and the results were obtained within 5 minutes. Samples with invalid results were tested with another test device. The results were immediately notified to the participants on the same day and then a satisfaction questionnaire about the screening program was completed. Individuals with a negative FIT result were recommended to be screened after 2 years. Participants who tested positive were referred to colonoscopy. For this purpose, CHWs conducted a pre-colonoscopy consultation to explain the procedure and bowel preparation, providing them with a prescription for bowel cleansing powders and an instructional pamphlet about colonoscopy, bowel preparation and the contact details of the endoscopy center. Participants were informed that they will receive a telephone call from the endoscopy center within four weeks to set a colonoscopy appointment.

 Colonoscopy appointments and patient instructions were conducted by a coordinator in the endoscopy center. If the individuals failed to undergo colonoscopy on the primary scheduled date, the coordinator made a follow-up call to offer a second appointment and ask them about the reasons for not undergoing a colonoscopy. Colonoscopies were performed at Shariati hospital in Tehran by two experienced endoscopists (having performed at least 200 procedures per year) under conscious sedation on pre-scheduled days every week. An anesthesiologist assessed the fitness of the participants for the procedure on the day of colonoscopy. Colonoscopy findings and any complications of the procedure were documented in a standardized report form.

 CHWs and the coordinator in the endoscopy center participated in an 8-hour training workshop, reviewing basic information on CRC and screening tests and study protocol in detail. They were encouraged to apply effective health communication to address the participant’s possible concerns regarding screening and to help them proceed with the program.

###  Financial Implications for the Participants

 More than 95% of Iranians have basic health insurance which covers 90% of healthcare expenditures made by public hospitals; however, medical services provided by the private health sector are not fully covered by the basic health insurance, where private insurance companies provide health insurance.^17^ Information about the screening costs was provided to the participants as part of the informed consent. The costs of FIT test, bowel preparation powders, and transport to the endoscopy center were paid for out of the study budget. The costs of colonoscopy, pathology samples, and cancer treatment were covered by patients depending on their insurance status.

###  Study Measures and Data Management

 Data management and entry were conducted by the research coordinator using an online operating designed software, Research Electronic Data Capture (REDCap) web application^18,19^ hosted at IARC. Participants’ demographics, knowledge and behavioral parameters, quality of bowel preparation and sedation, colonic lesion features (i.e., number, size, and location), and colonoscopy complications were documented. Advanced adenomas (AAs) were defined as adenomas sized ≥ 10 mm and/or with a villous component, and/or with high grade dysplasia, based on the pathological examinations. Detection rate was defined as the proportion of individuals detected with colonic neoplasms divided by the number of individuals having completed the FIT test, expressed per 1000 FIT-screened persons. The positive predictive value (PPV) of FIT test for AAs or CRC was calculated as the number of participants with AAs or CRC divided by the total number of participants who tested positive for FIT and underwent satisfactory colonoscopy. Incomplete colonoscopy procedures were excluded from the calculations. We surveyed the participants’ satisfaction with the entire FIT screening process, from stool collection at home to submission of samples at the PHCs and receipt of the screening results. The satisfaction questionnaire was developed and validated by specialists in gastroenterology and epidemiology who were involved in the study. Responses were measured using a four-point Likert scale (strongly agree, agree, disagree, strongly disagree). In addition, we investigated the causes of non-compliance with colonoscopy.

 Our primary outcomes included acceptance rate, kit return rate, FIT positivity, colonoscopy referral rate, colonoscopy completion, and adenoma, AAs and CRC detection rates, and reasons for non-compliance with colonoscopy. The secondary outcome pertained to the degree of satisfaction of the participants with the screening program.

###  Statistical Analysis

 We applied *t*-test and χ^2^ or Fisher’s exact tests for comparing means and proportions, respectively, reporting 95% confidence interval (CI) for estimates. Multivariable logistic regression was used to assess the adjusted effect of factors associated with colonoscopy compliance. Two-tailed tests were applied considering a *P* value of < 0.05 as statistically significant.

## Results

###  Participants’ Characteristics

**Figure 1:**
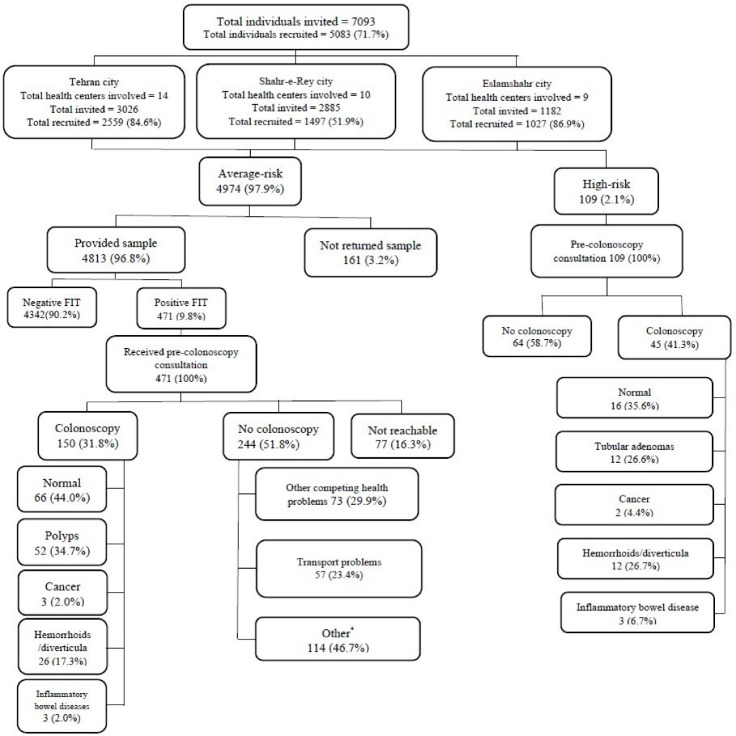
Recruitment flowchart *See Table 4

 The flow diagram of participant recruitment is shown in Figure 1. A total of 7039 individuals aged 50-75 years were invited through PHCs located in southern Tehran, Shahr-e-Rey and Eslamshahr. A total of 5083 (71.7%) invitees accepted to participate in the FIT-screening, out of whom 109 (2.1%) were considered high-risk and referred directly for colonoscopy and the remaining 4,974 average-risk participants received FIT kits between October 2018 and January 2019 (Figure 1).

 The mean age of the participants was 59 years (SD = 6.4), and a higher proportion were female (67.8%). Demographic characteristics of the participants by gender are shown in Table 1. Participants were invited mostly when attending PHCs for any reason (72.9%) or through telephone (27.1%). Most of the participants were married (84.5%), homemakers or unemployed (66.7%) and had primary-level or no formal education (71.2%) and basic health insurance (93.6%). The mass media, i.e. radio, television, and newspapers and the internet, were cited by 60.1% as the main source of medical information, 29.8% mostly trusted medical staff, and 10.1% relied on their relatives/friends. Only 1.3% of the participants had been previously screened for CRC (Table 1).

**Table 1:** Characteristics of the Participants by Gender

	**Male (n=1600)**	**Female (n=3374)**	**Both (n=4974)**
	**No. (%)**	**No. (%)**	**No. (%)**
Age (y)			
50–54	374 (23.3)	1029 (30.5)	1403 (28.2)
55–59	386 (24.1)	884 (26.2)	1270 (25.5)
60–64	382 (23.9)	825 (24.4)	1207 (24.3)
65–69	284 (17.8)	445 (13.2)	729 (14.7)
70–75	174 (10.9)	191 (5.7)	365 (7.3)
Registration for screening			
October-2019	109 (0.8)	205 (6.1)	314 (6.3)
November-2019	627 (39.2)	1359 (40.3)	1986 (39.9)
December-2019	579 (36.2)	1323 (39.2)	1902 (38.3)
January-2020	285 (17.8)	487 (14.4)	772 (15.5)
Invitation method			
In-person at health centers	1163 (72.7)	2462 (72.9)	3625 (72.9)
Phone call	437 (27.3)	912 (27.1)	1372 (27.1)
Marital status			
Married	1548 (96.8)	2655 (78.7)	4203 (84.5)
Single/divorced/widow/widower	52 (3.2)	719 (21.3)	771 (15.5)
Education			
No formal education/primary	921 (57.6)	2617 (77.5)	3538 (71.2)
Secondary	564 (35.2)	691 (20.5)	1255 (25.2)
University	115 (7.2)	66 (2.0)	181 (3.6)
Occupation			
Employed	73 (4.6)	42 (1.2)	115 (2.3)
Self-employed	691 (39.8)	55 (1.6)	692 (14.0)
Unemployed/homemaker	133 (8.3)	3184 (94.4)	3317 (66.7)
Retired	757 (47.3)	93 (2.8)	850 (17.0)
Health insurance			
None	82 (5.1)	236 (7.0)	318 (6.4)
Basic	1153 (72.1)	2446 (72.5)	3599 (72.4)
Basic plus private	365 (22.8)	692 (20.5)	1057 (21.2)
Main source for medical information			
TV/radio/Internet/newspapers	967 (60.4)	2024 (60.0)	2991 (60.1)
Medical staff	480 (30.0)	1000 (29.6)	1480 (29.8)
Relatives, friends, other	153 (9.5)	350 (10.4)	503 (10.1)
Prior CRC screening			
Yes	18 (1.1)	44 (1.3)	62 (1.3)
No	1582 (98.9)	3330 (98.7)	4912 (98.7)

###  Primary Outcomes

 A total of 4813 out of 4974 participants who were offered a FIT-kit returned their samples, a total uptake of 96.8%: with 84.6% (n = 4073) returning stool samples within 24 hours of receiving the FIT-kit. Among those who returned samples, only 77 participants (1.6%) had invalid test results for whom samples were re-tested with another device. FIT-testing on the participants showed a positive rate of 9.8% (n = 471). All participants with a positive FIT received pre-colonoscopy consultation and were referred for colonoscopy, of whom 21.9% (103/471) completed their colonoscopy as scheduled. Those who did not come for colonoscopy (n = 368) were contacted and 291 (79.1%) were reachable for a follow-up call and 47 (16.2%) of them underwent a colonoscopy afterwards. The overall compliance rate for colonoscopy was 31.8% (n = 150), with 30.7% (n = 46) having their colonoscopy completed within one month from the referral date. There was no statistically significant association between gender and screening and referral related variables (Table 2).

**Table 2:** Screening Process Measures and Intermediate Outcomes

	**Male**		**Female**		**Both**		***P*** ** Value**
	**No.**	**(%)**	**No.**	**(%)**	**No.**	**(%)**	
Participants registered	1600		3374		4974		
Participants who returned samples	1537	(96.1)	3276	(97.1)	4813	(96.8)	0.06
Returned within 24 h after receiving kit	1296	(84.3)	2777	(84.8)	4073	(84.6)	0.3
Returned 24–72 h after receiving kit	180	(11.7)	348	(10.6)	528	(11.0)	
Returned > 72 h after receiving kit	61	(4.0)	151	(4.6)	212	(4.4)	
Invalid FIT test results	30	(2.0)	47	(1.4)	77	(1.6)	0.4
One repeat test	28	(93.3)	44	(93.6)	72	(93.5)	
Two repeat tests	2	(6.7)	3	(6.4)	5	(6.5)	1.0
Participants with valid FIT results	1537		3276		4813		
Participants positive on FIT	152	(9.9)	319	(9.7)	471	(9.8)	0.9
Received pre-colonoscopy consultation	152	(100.0)	319	(100.0)	471	(100.0)	1.0
Colonoscopy completion after consultation	30	(19.7)	73	(22.9)	103	(21.9)	0.4
Participants targeted for follow-up call^a^	122		246		368		
Participants who responded follow-up call^b^	96	(78.7)	195	(79.3)	291	(79.1)	0.9
Colonoscopy completion after follow-up call	16	(16.7)	31	(15.9)	47	(16.2)	0.9
Overall compliance with colonoscopy	46	(30.3)	104	(32.6)	150	(31.8)	0.6
Poor bowel preparation^c^	2	(4.3)	4	(3.9)	6	(4.0)	0.9
Time between referral and colonoscopy completion							
Within 1 month	12	(26.1)	34	(32.7)	46	(30.7)	0.6
1-3 months	23	(50.0)	51	(49.0)	74	(49.3)	
> 3 months	11	(23.9)	19	(18.3)	30	(20.0)	

FIT, Fecal immunochemical test. ^a^ Participants who did not completed a colonoscopy after pre-colonoscopy consultation. ^b^ n = 77 were not reachable for the following reasons: phone numbers were no longer valid (n = 74), dead (n = 2), and moved (n = 1). ^c^ Among FIT positive participants with normal colonoscopy results.

 Overall, 16.8% (n = 835) received a reminder call to return the stool sample and 161 (3.2%) never returned their stool samples for the following reasons: they did not like stool sampling (77.0%), they were unable to collect samples (14.3%), and they did not have time to return samples (8.7%); there was no significant difference (*P*= 0.6) between genders regarding these reasons (data not shown).

 As reported in Table S1 (Supplementary File 1), polyps were detected in 34.7% (52/150), AAs in 13.3% (20/150), and cancer in 2.0% (3/150) of participants undergoing colonoscopy. The detection rates for CRC and AAs per 1000 FIT-screened participants were 0.6 (0.1–1.8) and 4.2 (2.5–6.4), respectively. The PPVs for CRC and AAs were 2.0% (0.4–5.7) and 13.3% (8.3–19.8), respectively (Table S2). The number of polyps per patient varied between 1–3 and 4–7, respectively, for 94.2% and 5.8% of patients detected to have polyps. The polyps measured less than 1 mm for the majority of the patients (67.3%) and ≥ 10 mm for 32.7%. Cecal intubation rate was 99.3% (n = 149/150) and in 4 patients (2.7%) colonoscopy was repeated due to inadequate bowel preparation within 1-month from the first procedure. There was no serious complication related to colonoscopies (data not shown).

 None of the demographic and background information were found to be associated with colonoscopy compliance in univariate analysis. We performed multivariable regression analysis including all the variables in the model which indicated no significant differences between participants who completed a colonoscopy and those who did not (Table 3).

**Table 3:** Determinants of Compliance to Colonoscopy among the FIT-Positive Participants

**Characteristics**	**FIT-Positive, n**	**Had Colonoscopy, No. (%)**	**Crude Odds Ratio (95% CI)**	**Adjusted Odds Ratio (95% CI)**
Participants registered	471	150 (31.9)		
Registration year				
2018	414	137 (33.1)	1.0 (Ref.)	1.0 (Ref.)
2019	57	13 (22.8)	0.6 (0.3–1.1)	0.6 (0.3–1.1)
Age (y)				
50–59	230	71 (30.9)	1.0 (Ref.)	1.0 (Ref.)
60–69	196	66 (33.7)	1.1 (0.7–1.7)	1.2 (0.7–1.8)
70–75	45	13 (28.9)	0.9 (0.6–1.3)	0.9 (0.6–1.3)
Gender				
Female	319	104 (32.6)	1.0 (Ref.)	1.0 (Ref.)
Male	152	46 (30.3)	1.1 (0.7–1.6)	0.9 (0.4–2.1)
Marital status				
Single/divorced/widowed	85	26 (30.6)	1.0 (Ref.)	1.0 (Ref.)
Married	386	124 (32.1)	1.0 (0.6–1.7)	1.1 (0.6–1.9)
Invitation method				
In-person	359	111 (30.9)	1.0 (Ref.)	1.0 (Ref.)
Phone call	112	39 (34.8)	1.1 (0.7–1.8)	1.1 (0.6–1.7)
Occupation				
No job, homemaker	322	105 (32.6)	1.0 (Ref.)	1.0 (Ref.)
Employed, retired	149	45 (30.2)	0.8 (0.5–1.3)	0.8 (0.3–1.7)
Education				
None, primary	366	115 (31.4)	1.0 (Ref.)	1.0 (Ref.)
Secondary and above	105	35 (33.3)	1.0 (0.9–1.1)	1.0 (0.9–1.1)
Medical insurance				
No	26	7 (26.9)	1.0 (Ref.)	1.0 (Ref.)
Yes	445	143 (32.1)	1.2 (0.5–3.1)	1.2 (0.4–2.9)
Kit return time after receiving				
Within 24 h	382	123 (32.2)	1.0 (Ref.)	1.0 (Ref.)
> 24 h	89	27 (30.3)	0.9 (0.5–1.5)	0.9 (0.5–1.5)
Source of medical information				
Mass media, Internet	296	96 (32.4)	1.0 (Ref.)	1.0 (Ref.)
Medical staff, relatives, friends	175	54 (30.9)	0.9 (0.6–1.3)	0.9 (0.6–1.3)
Prior CRC screening				
Yes	7	2 (28.6)	1.0 (Ref.)	1.0 (Ref.)
No	464	148 (31.9)	1.1 (0.2–6.1)	1.2 (0.2–6.7)

FIT, Fecal immunochemical test; CI, Confidence interval; Ref, Reference group.

 Reasons for non-compliance with colonoscopy among FIT-positive participants are shown in Table 4, which have been classified and presented as logistic barriers (e.g., time limits, scheduling challenges); health system obstacles (e.g., transport problems); and cognitive-emotional barriers (e.g., lack of perceived risk for CRC) (Table 4).

**Table 4:** Reasons for Non-compliance to Colonoscopy among FIT Positive Participants

	**Male (n=80)**	**Female (n=164)**	**Both (n=244)**^a^
	**No. (%)**	**No. (%)**	**No. (%)**
Logistic barriers			
Other health problems or more important worries	28 (35.0)	45 (27.4)	73 (29.9)
Time limits e.g., cannot have a day off from work	7 (8.8)	28 (17.1)	35 (14.3)
Health system barriers			
Transport problems/no escort to accompany	9 (11.3)	48 (29.3)	57 (23.4)
Doctor did not recommend a colonoscopy	13 (16.2)	15 (9.1)	28 (11.5)
CHWs are not competent	3 (3.8)	5 (3.1)	8 (3.3)
Cognitive-emotional barriers			
Being young or healthy makes getting cancer less likely	12 (15.0)	18 (11.0)	30 (12.3)
Colorectal cancer is incurable, screening is useless	7 (8.7)	2 (1.2)	9 (3.7)
Fear of the procedure and detecting cancer	1 (1.2)	3 (1.8)	4 (1.6)

FIT, fecal immunochemical test; CHWs, Community health workers. ^a^ n = 77 were not reachable for the following reasons: phone number was no longer valid (n = 74), dead (n = 2), and moved (n = 1).

 We measured the intervals (days) between: FIT kit collection and sample analysis, referrals and colonoscopy completion, and colonoscopy and final diagnosis according to the registration time, as shown in Table 5. The interval between kit collection and sample analysis showed a relatively stable pattern with time, with a median of one day throughout the study period. The interval between pre-colonoscopy consultation and colonoscopy completion increased with time, with median values ranging between 18.5–55.0 days and concomitantly compliance rate to colonoscopy showed a declining trend. Median values for the time interval between colonoscopy completion and final diagnosis verified by histopathology varied between 10.0–14.0 days (Table 5).

**Table 5:** Compliance to Colonoscopy and Intervals Between FIT Delivery, FIT Screening, Colonoscopy, and Final Diagnosis by Registration Period

	**Registration Period **	**Participants Assessed, n**	**Mean**	**SD (range)**	**Median (IQR)**	**Colonoscopy done, No. (%)**
Interval between FIT kit collection and FIT screening (days)	October 2018	310	1.4	2.6 (0.0-35.0)	1.0 (1.0-1.0)	NA
	November 2018	1926	1.4	1.6 (0.0-26.0)	1.0 (1.0-1.0)	NA
	December 2018	1839	1.3	1.5 (0.0-32.0)	1.0 (1.0-1.0)	NA
	January 2019	738	1.3	1.5 (0.0-32.0)	1.0 (1.0-1.0)	NA
	Total	4813	1.3	1.7 (0.0-35.0)	1.0 (1.0-1.0)	NA
Interval between pre-colonoscopy consultation and colonoscopy attendance among FIT positive patients (days)	October 2018	8	71.6	104.7 (5.0-267.0)	18.5 (7.0-125.0)	8/31 (25.8)
	November 2018	78	64.8	80.8 (2.0-394.0)	39.0 (22.0-61.0)	78/203 (38.4)
	December 2018	51	83.6	91.2 (1.0-357.0)	49.0 (27.0-78.0)	51/180 (28.3)
	January 2019	13	113.4	109.4 (12.0-344.0)	55.0 (35.0-180.0)	13/57 (22.8)
	Total	150	75.7	88.6 (1.0-394.0)	42.0 (22.0-66.0)	150/471 (31.8)
Interval between colonoscopy and final diagnosis among patients with pathology samples (days)	October 2018	3	15.0	8.9 (8.0-25.0)	12.0 (8.0-25.0)	NA
	November 2018	34	16.4	12.1 (5.0-62.0)	10.5 (8.0-21.0)	NA
	December 2018	17	16.8	12.5 (6.0-52.0)	10.0 (9.0-25.0)	NA
	January 2019	5	13.2	5.8 (7.0-21.0)	14.0 (8.0-16.0)	NA
	Total	59	16.2	11.5 (5.0-62.0)	10.0 (8.0-22.0)	NA

FIT: fecal immunochemical test; SD: standard deviation; IQR: interquartile range; NA: not applicable.

###  Secondary Outcomes

 Analysis of participants’ opinion and satisfaction level regarding the FIT-screening program indicated that at least 98.6% of the participants were satisfied with the time spent for FIT-testing and the communication and information provided by the CHWs. A remarkable number of participants (99.4%) mentioned that they would recommend FIT to their relatives and friends; 98.7% agreed to repeat FIT after 2 years if tested negative for FIT; 98.2% stated that a free test encouraged them to participate in screening; and 97.9% agreed with the necessity of undergoing a colonoscopy if they tested positive for FIT. CRC was cited as a concerning disease by 95.0% of the participants, 93.5% thought that FIT-testing was easy to do, 91.3% preferred to do the entire FIT-testing (sampling, buffer preparation and FIT reading) at home instead of returning stool samples to the PHCs, and 62.4% mentioned that stool collection was disgusting (Table S3).

## Discussion

 In the current study, FIT positivity rate was 9.8% with a cut-off value of 50 ng Hb/mL buffer, comparable to our previous study (9.1%) which used a quantitative FIT with a cut-off value of 100 ng Hb/mL.^11^ In Thailand, a qualitative FIT with a cut-off value of 200 ng Hb/mL led to a positivity rate of 1.1%, far below what was recorded in our study.^20^ The PPV of FIT for all adenomas per 100 FIT-positive participants was 8.7 in this study which is closely comparable to the respective value of 8.8 in our previous series.^11^ PPV varies depending on the method and the selected cut-off values of FIT^21,22^ and higher cut-off values may be even preferable in countries with limited-colonoscopy resources as it offers high PPV for advanced neoplasia and therefore reduces colonoscopy workload.^23^

 The desirable acceptance rate (96.8%) for FIT testing among our participants is comparable to the results (96.0%) of our previous pilot study.^11^ This could be partly explained by the effective health communication made by CHWs who are believed to build a personalized and trust-based relationship with the participants.^24-26^ Nevertheless, we recorded a low compliance for colonoscopy which was mainly due to the logistical and health system barriers, comparable to the results from other settings such as Morocco.^12,27^ In addition, we observed that with time, as the delay between referral to colonoscopy increased, compliance rate with colonoscopy declined. This means that the longer the waiting time for colonoscopy, the lower the compliance rate and the higher the loss-to-follow up. However, these long waiting times seem to be unavoidable, partly due to the increasing demand for colonoscopy associated with a screening program, and partly due to the limited capacity of the endoscopy center which is already limited for diagnostic procedures.

 Overall, implementing the first step of CRC screening seems to be feasible in Iran in both organizational and acceptance terms for the following reasons: the favorable test uptake for one-step FIT test, the simplicity of test application, and the well-established healthcare system staffed by trained CHWs.^24^ However, CRC screening is a multi-step process and Iran will be able to implement screening once the various components of a screening program will be readily available in place, e.g., insured budget at the Ministry of Health, development of screening guidelines and protocols, well-established referral system, improved diagnosis and treatment capacity in terms of staff and facilities, development of a health information system for monitoring, evaluations, and quality assurance. Furthermore, considering the recent challenges imposed by the effects of COVID-19, the low compliance rate for colonoscopy and the low incidence rate of CRC in Iran, we suggest the early diagnosis approach among symptomatic patients and high-risk groups enabling more efficient use of early detection and monitoring resources which eventually reduces unnecessary procedures in low-risk individual.^28^

 Our study has several limitations. First, we could not assess aspects such as cost-effectiveness and equity in the current feasibility study for FIT-screening, which should be thoroughly evaluated before FIT screening is introduced in a subsequent national programme. Second, our study sample was not representative of the general population as females comprised nearly 68.0% of the participants. This is explained by the fact that women visit PHCs more often than men; therefore, a system to invite men other than through PHC visits will be necessary.

 In conclusions,based on our results, FIT modality as a test of choice for CRC screening can be a safe and acceptable method of screening among the Iranian average-risk population. However, the suboptimal compliance rate with colonoscopy outweighs the advantages of FIT screening. We therefore suggest improving awareness in the general population with an early diagnosis approach among symptomatic patients and high-risk individuals. The results of the current study may not be limited to Iranians and could have implications for other developing countries with similar trends in the CRC epidemic.

## Supplementary File

Supplementary file 1 contains Tables S1-S3.
