# Supplementary material for: Colorectal Cancer Screening Pilot Project in Tehran-Iran, a Feasibility Study
Source: Arch Iran Med. 2023 Mar 1;26(3):138–46. doi: 10.34172/aim.2023.22 (PMC10685729; doi:10.34172/aim.2023.22)
Supplement: Supplementary file 1 — contains Tables S1-S3. [file aim-26-138-s001.pdf]

Arch Iran Med. March 2023;26(3):138-146
10.34172/aim.2023.22
http://journalaim.com

Supplementary file 1

Table S1. Colonoscopy and histopathology outcomes among FIT positive participants

	**Male (*n*=46)**	**Female, (*n*=104)**	**Both, (*n*=150)**	***P*-Value**
	***n* (%)**	***n* (%)**	***n* (%)**	
Normal	18 (39.1)	48 (46.2)	66 (44.0)	0.6
Polyps	18 (39.1)	34 (32.7)	52 (34.7)	
Suspicious cancer	1 (2.2)	2 (1.9)	3 (2.0)	
Hemorrhoids/diverticula	7 (15.2)	19 (18.2)	26 (17.3)	
Inflammatory bowel diseases	2 (4.4)	1 (1.0)	3 (2.0)	
Investigations*				
Biopsy	5 (10.9)	3 (2.9)	8 (5.3)	0.05
Polypectomy	18 (39.1)	34 (32.7)	52 (34.7)	0.6
Histopathology				
Inflammatory/hyperplastic polyps	2 (4.4)	4 (3.8)	6 (4.0)	0.4
Tubular adenomas	16 (34.8)	25 (24.0)	41 (27.3)	
Advanced adenomas	9 (19.6)	11 (10.6)	20 (13.3)	
Colorectal cancer	1 (2.2)	2 (1.9)	3 (2.0)	
Polyps with unknown pathology	0 (0.0)	5 (4.8)	5 (3.3)	

FIT, Fecal immunochemical test; *among participants positive on FIT with abnormal colonoscopy.

**Table S2.** Detection rates and PPV of FIT for detecting colonic neoplasms					
	**Cases detected**	**FIT returned**	**Colonoscopy done**	**Detection rate***	**PPV (%) ****
Adenomas					
Female	25	3276	104	7.6 (4.9–11.2)	24.0 (16.2–33.4)
Male	16	1537	46	10.4 (5.9–16.8)	34.8 (21.3–50.2)
Total	41	4813	150	8.5 (6.1–11.5)	27.3 (20.4–35.2)
Advanced adenomas					
Female	11	3276	104	3.4 (1.7–6.0)	10.6 (5.4–18.1)
Male	9	1537	46	5.9 (2.6–11.0)	19.6 (9.4–33.9)
Total	20	4813	150	4.2 (2.5–6.4)	13.3 (8.3–19.8)
Colorectal cancer					
Female	2	3276	104	0.6 (0.07–2.0)	1.9 (0.2–6.8)
Male	1	1537	46	0.7 (0.01–3.6)	2.2 (0.06–11.5)
Total	3	4813	150	0.6 (0.1–1.8)	2.0 (0.4–5.7)
Advanced adenomas and colorectal cancer					
Female	13	3276	104	4.0 (2.1–6.7)	12.5 (6.8–20.4)
Male	10	1537	46	6.5 (3.1–11.9)	21.7 (10.9–36.4)
Total	23	4813	150	4.8 (3.0–7.1)	15.3 (9.9–22.1)
PPV, Positive predictive value; FIT, Fecal immunochemical test; *per 1000 FIT screened participants, **per 100 colonoscopies done.					

**Table S3:** Participant's opinion and satisfaction level regarding FIT testing (n=4818)				
	**Strongly agree**	**Agree**	**Disagree**	**Strongly disagree**
I am satisfied with time spent for FIT testing	3646 (75.7)	1104 (22.9)	60 (1.2)	8 (0.2)
I am satisfied with CHWs communication	3781 (78.5)	1035 (21.5)	1 (0.02)	1 (0.02)
I am satisfied with information given by CHWs	3745 (77.7)	1070 (22.2)	2 (0.04)	1 (0.02)
I would recommend FIT to my relatives and friends	3471 (72.1)	1317 (27.3)	26 (0.5)	4 (0.1)
With a negative FIT, it is necessary to do FIT after 2 years	3432 (71.2)	1324 (27.5)	57 (1.2)	5 (0.1)
Free test encourages me to participate	3497 (72.6)	1232 (25.6)	77 (1.6)	12 (0.2)
With a positive FIT, it is necessary to complete a colonoscopy	3396 (70.5)	1321 (27.4)	92 (1.9)	9 (0.2)
Colorectal cancer is a concerning disease	3085 (64.1)	1489 (30.9)	209 (4.3)	35 (0.7)
FIT testing is easy to do	3136 (65.1)	1368 (28.4)	243 (5.0)	71 (1.5)
I prefer to do FIT testing at home	3099 (64.3)	1300 (27.0)	163 (3.4)	256 (5.3)
Stool collection is disgusting	1908 (39.6)	1100 (22.8)	1291 (26.8)	519 (10.8)
FIT: fecal immunochemical test, CHW: community health workers				
